# Prenatal dietary load of Maillard reaction products combined with postnatal Coca-Cola drinking affects metabolic status of female Wistar rats

**DOI:** 10.3325/cmj.2015.56.94

**Published:** 2015-04

**Authors:** Radana Gurecká, Ivana Koborová, Katarína Janšáková, Tamás Tábi, Éva Szökő, Veronika Somoza, Katarína Šebeková, Peter Celec

**Affiliations:** 1 Institute of Molecular Biomedicine, Faculty of Medicine, Comenius University, Bratislava, Slovakia; 2 Department of Pharmacodynamics, Faculty of Pharmacy, Semmelweis University, Budapest, Hungary; 3 Department of Nutritional and Physiological Chemistry, Faculty of Chemistry, University of Vienna, Vienna, Austria

## Abstract

**Aim:**

To assess the impact of prenatal exposure to Maillard reaction products (MRPs) -rich diet and postnatal Coca-Cola consumption on metabolic status of female rats. Diet rich in MRPs and consumption of saccharose/fructose sweetened soft drinks is presumed to impose increased risk of development of cardiometabolic afflictions, such as obesity or insulin resistance.

**Methods:**

At the first day of pregnancy, 9 female Wistar rats were randomized into two groups, pair-fed either with standard rat chow (MRP-) or MRPs-rich diet (MRP+). Offspring from each group of mothers was divided into two groups and given either water (Cola-) or Coca-Cola (Cola+) for drinking *ad libitum* for 18 days. Oral glucose tolerance test was performed, and circulating markers of inflammation, oxidative stress, glucose and lipid metabolism were assessed.

**Results:**

MRP+ groups had higher weight gain, significantly so in the MRP+/Cola- vs MRP-/Cola-. Both prenatal and postnatal intervention increased carboxymethyllysine levels and semicarbazide-sensitive amine oxidase activity, both significantly higher in MRP+/Cola + than in MRP-/Cola-. Total antioxidant capacity was lower in MRP+ groups, with significant decrease in MRP+/Cola + vs MRP-/Cola+. Rats drinking Coca-Cola had higher insulin, homeostatic model assessment of insulin resistance, heart rate, advanced oxidation of protein products, triacylglycerols, and oxidative stress markers measured as thiobarbituric acid reactive substances compared to rats drinking water, with no visible effect of MRPs-rich diet.

**Conclusion:**

Metabolic status of rats was affected both by prenatal and postnatal dietary intervention. Our results suggest that combined effect of prenatal MRPs load and postnatal Coca-Cola drinking may play a role in development of metabolic disorders in later life.

Maillard reaction products (MRPs), first described by French biochemist C. Maillard in the beginning of 20th century (1), are formed by nonenzymatic reactions of reactive sugars and proteins, giving thermally processed food its typical color, taste, and odor.

Eight decades later Brownlee et al. recognized that same substances are formed naturally in human body, and named the *in vivo* analogues of MRPs “advanced glycation end products” (AGEs) (2,3). Except for classical pathway of their formation under hyperglycemic conditions, there are alternative pathways of AGEs formation effective – under oxidative- and carbonyl-stress, utilizing reactive aldehydes formed during lipid peroxidation and autooxidation of glucose. AGEs are implicated in pathophysiology of aging and different non-communicable diseases: AGE-modification alters the structure (physical and chemical properties) and thus function (biological properties) of proteins (4). Discovery of specific cell-surface receptor for AGEs (RAGE) enabled characterization of indirect harmful pathways leading to enhanced oxidative stress and pro-inflammatory, diabetogenic, and atherogenic effects (5,6).

In 1997, Koschinsky et al (7) showed that dietary MRPs partially absorbed into the bloodstream were chemically and biologically active, exerting harmful health effects, which is why they were called “glycotoxins.” This finding prompted extensive research confirming that consumption of large amounts of dietary MRPs might induce or aggravate insulin resistance, renal impairment or atherosclerosis, activate inflammatory and oxidative stress pathways, and contribute to development of complications in diabetes and nephropathies (8-11). These findings raise the question on the role of MRPs-rich diet in prenatal programming. Evidence strongly suggests that maternal obesity and improper prenatal nutrition provide maladaptive intrauterine cues to developing offspring, predisposing organs for chronic disease later in life (12,13). Maternal dietary habits affect the fetus, outcome of pregnancy, and long term health of the child (14-16). Mericq et al found a direct relationship between newborn’s and maternal serum levels of several AGEs at the time of delivery, suggesting maternal transmission of AGEs (17). AGEs/RAGE axis activates in pregnancy-associated pathologies impacting fetus development, such as preeclampsia and preterm birth (18-20).

Rising prevalence of obesity and obesity-associated (particularly metabolic) complications in youth (21,22) was linked, among others, to rising consumption of sugar-sweetened carbonated drinks such as cola beverages (23,24). Effects are attributed to multiple factors, including higher caloric intake, high fructose content rendering less satiety and compensation and resulting in elevated plasma uric acid, and a general effect of consuming refined carbohydrates (25,26). Moreover, cola beverages also contain MRPs and reactive AGE-precursors, most abundantly hydroimidazolone derived from arginine residues modified by methylglyoxal (27,28).

To the best of our knowledge potential effects of MRPs-rich diet during pregnancy on prenatal programming have yet not been investigated. In this study we investigated the metabolic status of young adult rats – offspring of mothers consuming MRPs-rich diet during pregnancy. As drinking of cola beverages is increasingly popular among children and adolescents, our second aim was to investigate the additional impact of Coca-Cola consumption on prenatally affected young adult rats.

## Material and methods

### Animals

The study was conducted according to the guidelines for experimental studies using laboratory animals (86/609/EEC) and approved by the institutional ethics committee (number 025/2013/UPF, 6.6.2013). Female Wistar rats obtained from AnLab (Prague, Czech Republic) were housed under controlled room temperature and humidity, with 12 hours/12 hours light-dark cycle.

### Experimental design

At the first day of pregnancy, 9 rats were randomized into 2 groups (n = 4-5) and pair-fed with either standard rat chaw or chow enriched with bread crusts as a source of MRPs (bread crusts: standard rat chow 25%:75% wt/wt) until delivery. Bread crusts from German sourdough bread were prepared as described previously (29). Consumption of standard chow in the control group was recorded daily and the same amount of MRPs-rich diet was given to the experimental group on the following day.

**Figure 1:**
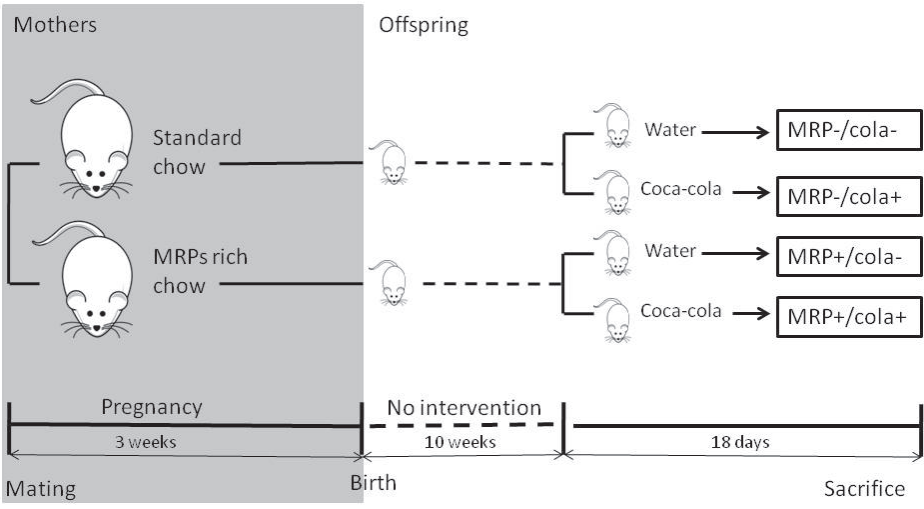
Diagram of the study design. MRPs – Maillard reaction products.

At the age of 10 weeks, female offspring from each group were divided into two weight- matched groups (n = 10-15). Both groups were fed with standard rat chow and were given either water or decarbonated Coca-Cola (sugar 110 g/L, caffeine 100 mg/L, energy 1800 kJ/L) for drinking *ad libitum*. Thus, the study included the following 4 groups: MRP-/Cola- (standard chow/water drinking); MRP+/Cola- (MRPs-rich diet/water drinking); MRP-/Cola+ (standard chow/Coca-Cola drinking; MRP+/Cola+ (MRPs-rich diet/Coca-Cola drinking). The animals were sacrificed after 18 days of intervention (Figure 1).

Two days before sacrifice, systolic blood pressure and heart rate were measured by noninvasive tail-cuff plethysmography (Hugo-Sachs Elektronik, Freiburg, Germany) and one day before sacrifice oral glucose tolerance test was performed. After overnight fasting with water or Coca-Cola available *ad libitum*, rats were administered 2 g/kg body weight of glucose dissolved in 0.5 mL of water via gavage. Blood glucose levels were measured using standard glucose meter in blood from the tail, before glucose administration and 15, 30, 60, 90 and 120 minutes thereafter. Animals were sacrificed after overnight fasting with water or Coca-Cola available *ad libitum*. Urine from bladder and blood samples from the abdominal aorta (serum and K3EDTA plasma) were collected under i.p. ketamin/xylazin anesthesia. Samples were aliquoted and stored frozen until analysis.

### Biochemical analysis

Albumin, total cholesterol, and triacylglycerols concentration and aspartate transaminase (AST) activity were analyzed by standard methods using autoanalyzer. Fasting insulin was measured using Rat Ultrasensitive Insulin ELISA (ALPCO Diagnostics, Salem, MA, USA). To assess carbonyl stress in the samples, AGEs-associated fluorescence of plasma was measured spectrofluorometrically (30), fructosamine was measured as described elsewhere (31), and Nϵ-carboxymethyllysine (CML) with AGE-CML ELISA kit (Microcoat, Bernried am Starnberger See, Germany). To assess oxidative damage to proteins, advanced oxidation protein products (AOPP) were determined using the spectrophotometric method described by Witko-Sarsat et al (32) and modified by Anderstam (33), and thiobarbituric acid reactive substances (TBARS) were measured spectrofluorometrically (34). The antioxidant status was assessed using two assays – ferric reducing antioxidant power (FRAP) (35) and total antioxidant capacity (TAC) (36). Activity of semicarbazide-sensitive amine oxidase (SSAO) was determined by radiometric method (37). Urinary creatinine was measured using the spectrophotometric method by Jaffe (38) and proteins were quantified using BCA protein assay kit (Sigma Aldrich, Steinheim, Germany). All measurements were performed using Saphire II multi-mode plate reader (Tecan, Grödig, Austria), and chemicals and reagents used were purchased from Sigma-Aldrich.

AGE-associated fluorescence of plasma, CML, AOPP, and SSAO activity were normalized to serum albumin. Homeostatic model assessment of insulin resistance (HOMA-IR) was calculated using the formula: fasting insuline (μIU/mL) × fasting plasma glucose (mmol/L)/22.5. Area under glucose curve was calculated from the OGTT data. Urinary albumin/creatinine ratio was also calculated.

### Statistical analysis

Data are presented as mean ± standard deviation (SD) for variables with nonparametric distribution written in italics. On figures, data are presented as minimum, first quartile, median, third quartile and maximum. For comparison between groups of data with normal distribution, one-way ANOVA test with subsequent Tukey's multiple comparison test was used. For data with non-normal distribution, Kruskal-Wallis test with subsequent Dunn's multiple comparison test was used. *P* values <0.05 were considered significant. Data were analyzed using GraphPad Prism 6.0 (GraphPad Software, La Jolla, CA, USA).

## Results

### Pregnancy outcomes

MRPs + and MRPs- rats did not differ significantly in weight gain during pregnancy (86 ± 24 g and 81 ± 25 g, respectively, *P* = 0.670) or in the number of pups delivered (10 ± 3 and 11 ± 2, respectively, *P* = 0.517). Since MRPs + mothers delivered significantly greater number of female pups (30 female/11 male in MRP+; 21 female/25 male in MRP-; *P* = 0.016), female offspring were used in our experiment.

### Metabolic study in female offspring

Metabolic status of offspring was affected both by prenatal MRPs load and postnatal Coca-Cola drinking, some of the effects occurred only as a result of combined intervention (Table 1).

**Table 1:** Overview of results and statistical analysis – ANOVA respectively Kruskal-Wallis test and post hoc comparison between all pairs of groups. Data are given as mean ± standard deviation, for variables with nonparametric distribution written in italics. *P* values <0.05 were considered significant, in bold

	MRP-/ Cola- n = 10	MRP+/ Cola- n = 15	MRP-/ Cola+ n = 11	MRP+ /Cola+ n = 15	ANOVA resp. Kruskal-Wallis	MRP-/ Cola-vs MRP+/ Cola-	MRP-/ Cola- vs MRP-/ Cola+	MRP-/ Cola- vs MRP+/ Cola+	MRP+/ Cola- vs MRP-/ Cola+	MRP+/ Cola-vs MRP+/ Cola+	MRP-/ Cola+ vs MRP+/ Cola+
Fluid consumption (/d/rat)	*19.8* *±3.6*	*17.3* *±1.4*	*72.0* *±5.0*	*63.2* *±14.4*	***<0.001***	*>0.999*	***0.002***	***<0.001***	***<0.001***	***<0.001***	*>0.999*
						
Initial weight (g)	*156* *±40*	*119* *±30*	*159* *±42*	*120* *±32*	***0.043***	*0.414*	*>0.999*	*0.303*	*0.240*	*>0.999*	*0.169*
						
Blood pressure (mmHg)	*141* *±19*	*123* *±10*	*125* *±12*	*127* *±20*	*0.057*						
						
Heart rate	447 ±49	429 ±67	501 ±28	485 ±53	**0.004**	0.848	0.101	0.293	**0.007**	**0.028**	0.876
						
Insulin (mIU/L)	*2.99* *±2.39*	*2.22* *±1.65*	*13.29* *±7.26*	*8.80* *±4.75*	***<0.001***	*>0.999*	***0.001***	***0.030***	***<0.001***	***0.003***	*>0.999*
						
HOMA-IR	*0.600* *±0.453*	*0.588* *±0.484*	*3.355* *±1.736*	*2.536* *±1.270*	***<0.001***	*>0.999*	***<0.001***	*0.009*	***<0.001***	*0.003*	*>0.999*
						
Cholesterol (mmol/L)	1.62 ±0.27	1.61 ±0.31	1.85 ±0.33	1.77 ±0.34	0.245						
						
*TAG (mmol/L)*	*0.60* *±0.21*	*0.59* *±0.18*	*1.44* *±0.64*	*1.20* *±0.67*	***<0.001***	*>0.999*	***0.008***	***0.012***	***0.006***	***0.008***	*>0.999*
						
AST activity (ukat/L)	1.66 ±0.33	1.90 ±0.44	1.82 ±0.33	1.56 ±0.26	0.076						
						
AGEs fluorescence (AU/g alb)	*278* *±47*	*281* *±99*	*246* *±64*	*322* *±127*	*0.156*						
						
Frustosamine (mmol/L)	*1.49* *±0.38*	*1.64* *±0.24*	*2.39* *±0.77*	*2.51* *±0.68*	***<0.001***	*>0.999*	***0.009***	***0.003***	*0.060*	***0.017***	*>0.999*
						
TBARS (umol/)	*5.90* *±0.83*	*5.10* *±0.72*	*6.74* *±0.75*	*6.91* *±1.15*	***<0.001***	*0.142*	*0.144*	***0.034***	***<0.001***	***<0.001***	*0.964*
						
FRAP (umol/)	165.3 ±28.2	179.1 ±32.5	225.0 ±47.5	216.9 ±59.4	0.005	0.869	**0.017**	**0.035**	0.057	0.114	0.969
						
Urine alb/crea (mg/mmol)	*41.8* *±24.8*	*33.2* *±20.8*	*57.6* *±30.4*	*59.0* *±48.9*	*0.157*						
						

MRP – Maillard reaction products; HOMA-IR – model assessment of insulin resistance; TAG – triacylglycerols; AST – aspartate transaminase; AGEs – advanced glycation end products; TBARS – thiobarbituric acid reactive substances; FRAP – ferric reducing antioxidant power; alb/crea – albumine to creatinine ratio.

### Fluid consumption and weight gain

**Figure 2:**
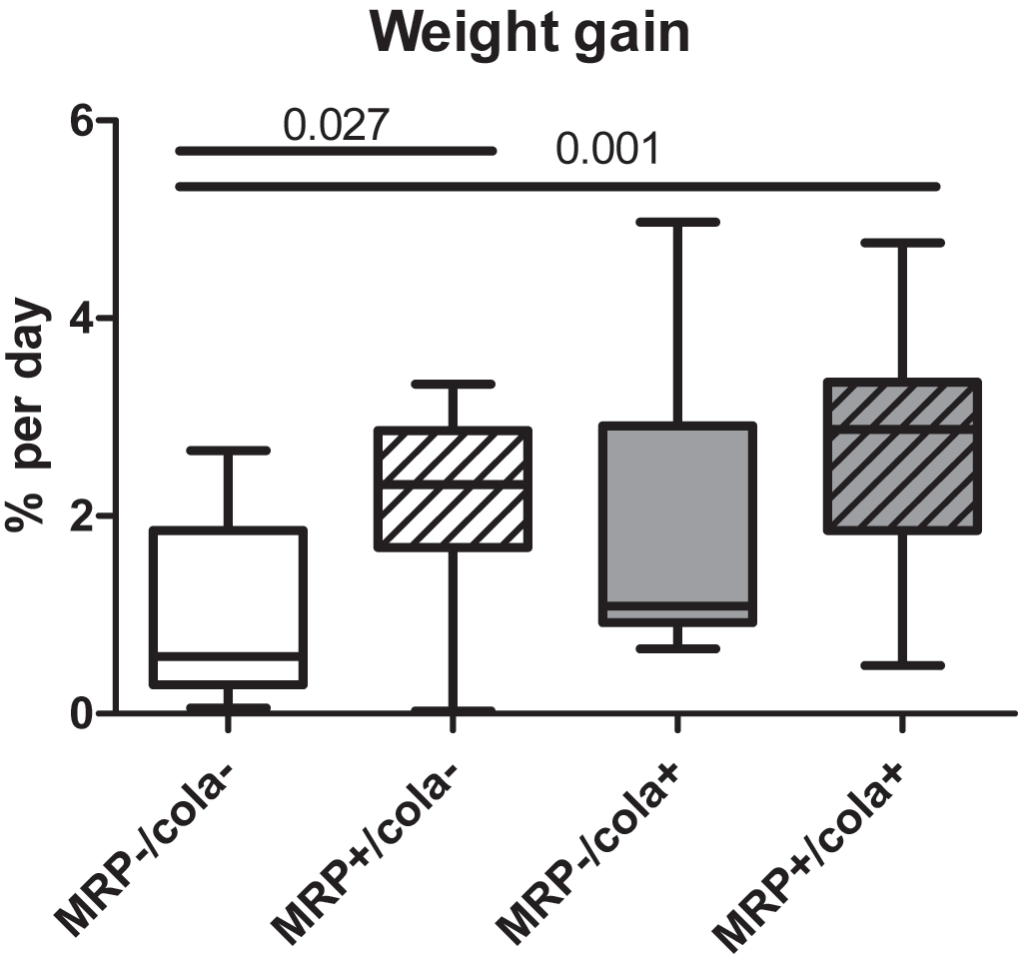
Relative weight gain was increased by prenatal intervention with Maillard reaction products (MRP) or its combination with Coca-Cola intake, but not Coca-Cola intake itself. Data were presented as minimum, first quartile, median, third quartile and maximum. Kruskal-Wallis test with subsequent Dunn's multiple comparison test to compare all pairs of groups were used. Significant differences between the groups were shown.

Fluid consumption was significantly higher in Coca-Cola drinking groups than in water drinking groups (Table 1). Despite similar weight at the beginning of the intervention (Table 1), higher weight gain was observed in MRP+ groups, but the increase was significant only in the group drinking water (Figure 2).

### Blood pressure and heart rate

Blood pressure did not differ significantly between the groups and heart rate was slightly higher in Coca-Cola drinking groups (Table 1).

### Glucose metabolism

**Figure 3:**
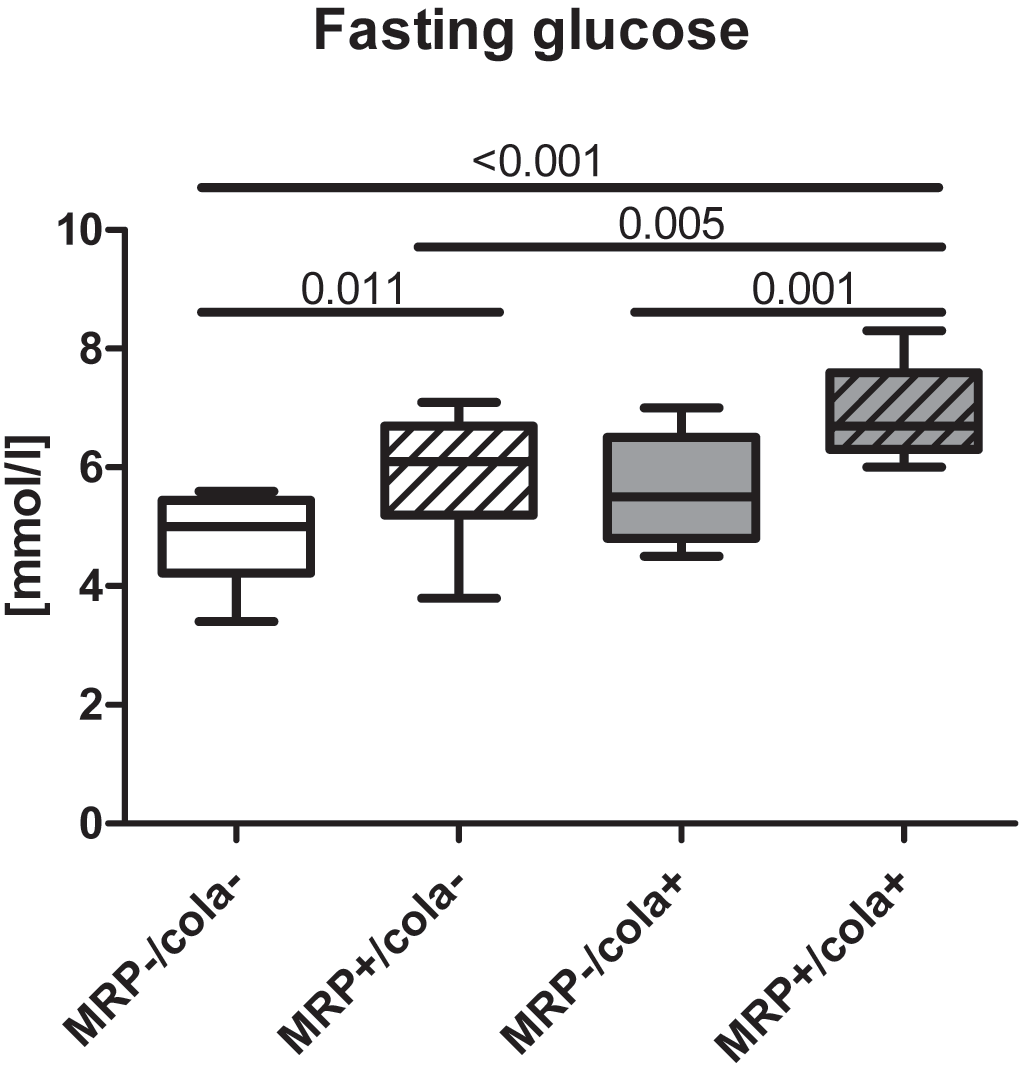
Fasting glucose levels were increased by prenatal Maillard reaction products (MRP) diet and by its combination with Coca-Cola intake. Data were presented as minimum, first quartile, median, third quartile and maximum. ANOVA test with subsequent Tukey's multiple comparison test to compare all pairs of groups were used. Significant differences between the groups were shown.

**Figure 4:**
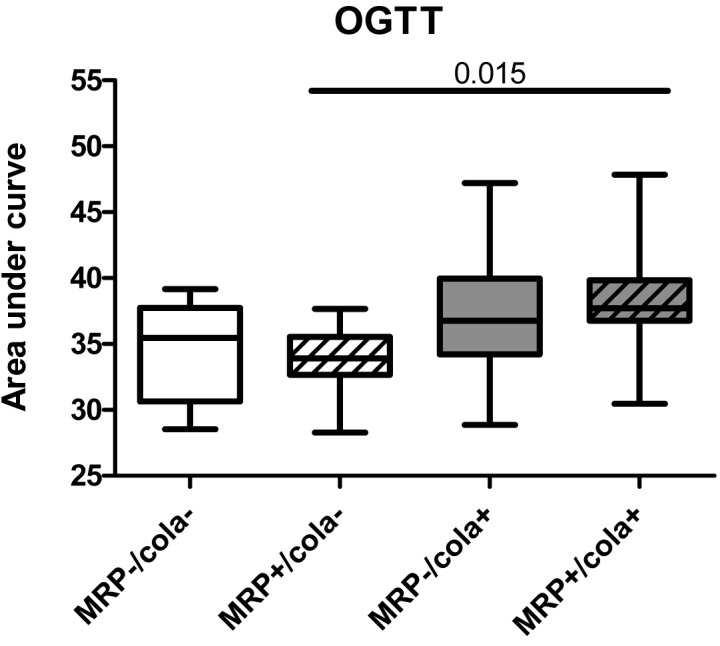
The area under curve during oral glucose tolerance test (OGTT) was slightly greater in Coca-cola drinking groups, but the effect was significant only in groups prenatally exposed to Maillard reaction products (MRP). Data were presented as minimum, first quartile, median, third quartile and maximum. ANOVA test with subsequent Tukey's multiple comparison test to compare all pairs of groups were used. Significant differences between the groups were shown.

Fasting glucose levels were significantly higher in both MRP+ groups than in MRPs- groups. A significant increase was noticed in Coca-Cola drinking groups (Figure 3). Both insulin concentration and insulin resistance, expressed as HOMA-IR, were higher in Coca-Cola drinking groups than in water drinking groups, regardless of prenatal intervention (Table 1). The area under glucose curve during OGTT was slightly greater in Coca-Cola drinking groups, but the increase was significant only between MRP+/Cola- and MRP+/Cola+ (Figure 4).

### SSAO activity

**Figure 5:**
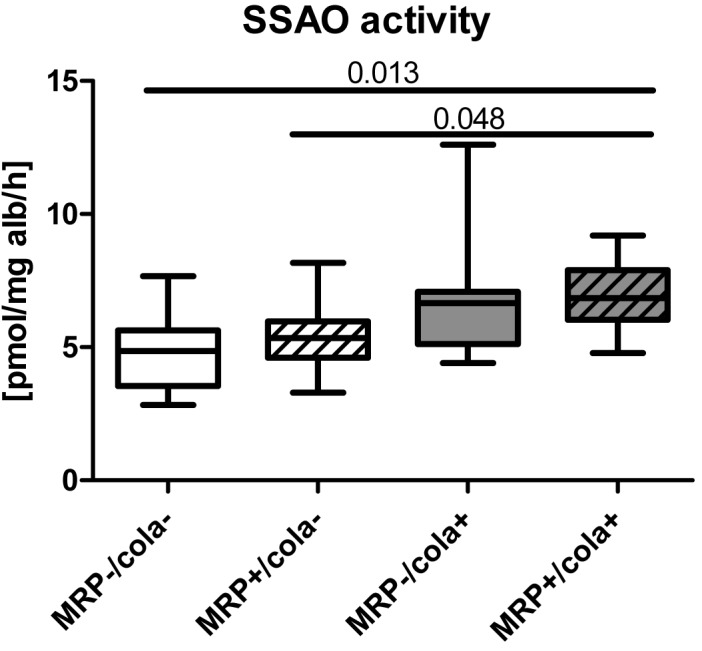
Activity of semicarbazide-sensitive amine oxidase (SSAO) was increased by the combined intervention of prenatal Maillard reaction products (MRP) intake and postnatal Coca-Cola intake. Kruskal-Wallis test with subsequent Dunn's multiple comparison test to compare all pairs of groups were used. Significant differences between the groups were shown.

Higher SSAO activity was found in MRP+ /Cola + group than in MRP-/Cola- and MRP+/Cola- group (Figure 5).

### Lipid metabolism and AST

Total cholesterol was not affected by any of the interventions; higher levels of triacylglycerols were found in Coca-Cola drinking groups, regardless of prenatal intervention. No differences in AST activity were observed between groups (Table 1).

### Carbonyl stress

**Figure 6:**
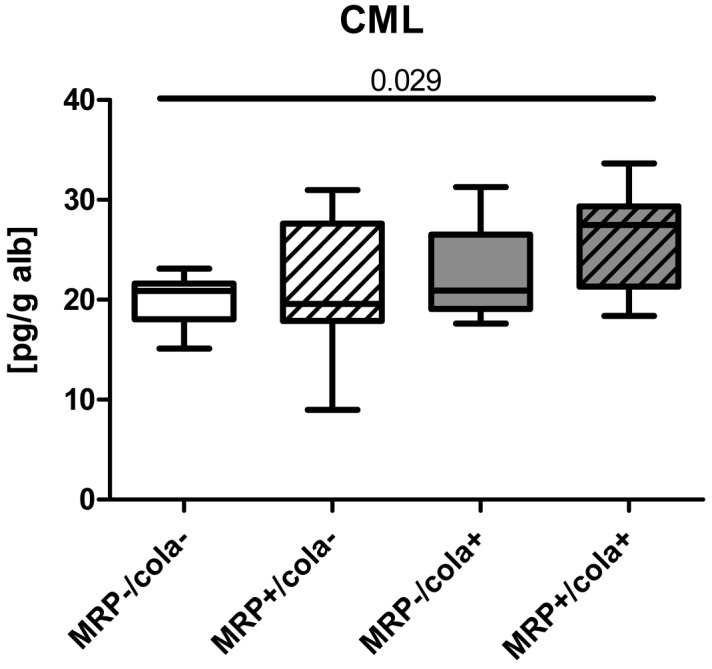
Circulating levels of carboxymethyllysine (CML) were increased by the combined intervention of prenatal Maillard reaction products (MRP) intake and postnatal Coca-Cola intake. ANOVA test with subsequent Tukey's multiple comparison test to compare all pairs of groups were used. Significant differences between the groups were shown.

AGE-associated fluorescence of plasma did not differ significantly between the groups (Table 1). Higher plasma CML/Alb was found in MRP+/Cola + group than in MRP-/Cola- group (Figure 6). Higher concentrations of fructosamine were found in Coca-Cola drinking groups (Table 1).

### Oxidative status

**Figure 7:**
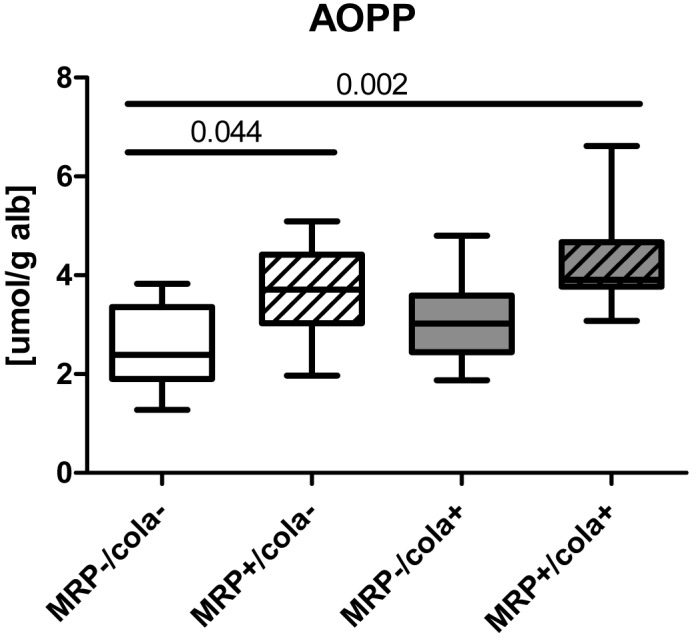
Plasma advanced oxidation protein products-to-albumine ratio (AOPP/Alb) was significantly increased by prenatal challenge of Maillard reaction products (MRP). Kruskal-Wallis test with subsequent Dunn's multiple comparison test to compare all pairs of groups were used. Significant differences between the groupswere shown.

**Figure 8:**
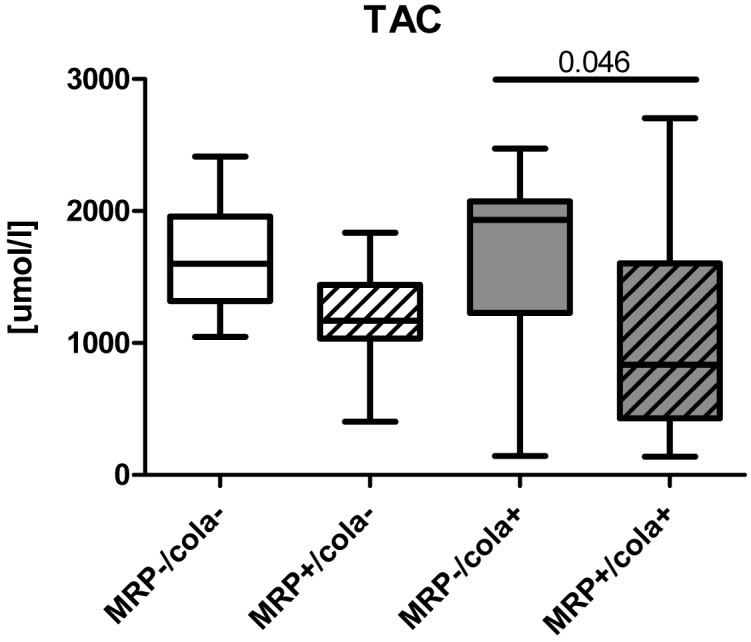
Total antioxidative capacity (TAC) was decreased by prenatal load of Maillard reaction products (MRP) with a significant decrease in rats drinking Coca-Cola. Significant differences between the groups are shown.

Plasma AOPP/Alb was significantly higher in MRPs + than in MRPs- groups (Figure 7). TBARS concentration was increased in Coca-Cola drinking groups, with significant increase in MRP+/Cola + group (Table 1). TAC was lower in MRP+ groups, with significant decrease in MRP+/Cola + group (Figure 8). FRAP was higher in Coca-Cola drinking groups (Table 1).

### Renal function

Urinary albumin-to-creatinine ratio did not differ significantly between the groups (Table 1).

## Discussion

To the best of our knowledge, this is the first study investigating the effect of maternal diet rich in MRPs together with post-natal effect of drinking Coca-Cola on the metabolic profile of the offspring. Our results suggested that intrauterine exposure to MRP-rich diet resulted in certain metabolic alterations in the offspring, rendering them susceptible to the effects of sweetened soft beverages. Thus, pre-natal intervention resulted in weight gain, impaired glucose homeostasis, and increased AOPP levels, and postnatal drinking of Coca-Cola further impaired glucose homeostasis, elevated plasma CML levels, increased plasma activity of SSAO, and altered oxidative status.

Studies dealing with the impact of maternal diet on health status of the offspring employ generally either undernutrition or overnutrition models. Epidemics of obesity has been associated with consumption of Western diet rich in fat and saccharides. High fat content in the diet of mothers was shown to have negative effects on offspring’s health in mice (39) and rats (40), as well as in humans (41). However, a Western diet is not a general equivalent of MRP-rich diet: if boiled or steamed, rise in MRPs is negligible in comparison with frying, broiling, or roasting (10,28). Thus, to study the effect of oral MRP load we used bread crusts-enriched diet, as no fat is added to bread dough and it is baked under high temperatures (220°C-260°C) (29). Consumption of analogous MRP-rich diet by adult rats in our previous experiments was associated with rise in circulating and tissue AGEs, metabolic alterations including diabetogenic effects, and nephrotoxicity (42-44). Since we wanted to eliminate the metabolic effects of different amounts of consumed proteins (45), the rats in our study were pair-fed.

### Prenatal MRPs-rich diet effects

In this study, prenatal MRPs load lead to higher weight gain in young adult rats. In our former experiment, adult rats on MRPs-rich diet gained more weight than rats on a standard rat chow, despite pair-feeding (43). The effect of MRPs-rich diet *in utero* on postnatal weight gain of the offspring deserves verification in further studies. A large prospective study in humans showed that maternal diet was associated with body composition of their adolescent offspring (41).

In our study, prenatal intervention was associated with impaired glucose homeostasis of the offspring. This finding corresponded with the data showing that a frequent fried food (generally rich in MRPs) consumption before pregnancy was significantly associated with a greater risk of incident gestational diabetes mellitus (46). Moreover, correlations between serum levels of certain AGEs and serum insulin and HOMA of 12-month-old infants were observed (17).

AOPPs are formed by myeloperoxidase reaction, pointing to enhanced activity of phagocytes. In contrast to our previous study in which 3 weeks-long MRPs rich diet was not associated with significant change in AOPP levels (42), offspring in this study subjected to prenatal MRPs load had higher AOPPs than the offspring subjected to standard rat chow. These results definitely should be confirmed by other studies.

### Effects of combined pre- and post-natal challenge

Administration of Coca-Cola to young adult rats was selected to mimic the dietary pattern typical for youth in western countries. However, since the metabolic effects of consumption of cola beverages by adult rats have already been studied (47), we particularly focused on the impact of the combined pre- and post-natal dietary interventions.

In our study, following prenatal exposure to MRPs, the offspring were more sensible susceptible to the impact of sweetened beverage consumption on glucose homeostasis, which was not evident in groups without prenatal intervention. These data were in accordance with the finding that after 3-month long administration of Coca-Cola, rats did not show changes in glucose metabolism; drinking Pepsi-Cola was even associated with lower HOMA-IR (47). Thus, prenatal challenge with high dietary MRPs load might negatively modulate the response to high saccharide consumption in the form of saccharose/fructose beverages.

SSAO represents a group of heterogeneous enzymes converting primary amines such as methylamine and aminoacetone, into corresponding aldehydes (eg, formaldehyde and methylglyoxal, respectively). Reactive aldehydes are generally toxic, eg, methylglyoxal, a precursor of AGEs, is, among others, implicated in pathogenesis of insulin resistance (48,49). This enzymatic reaction also produces hydrogen peroxide, inducing or aggravating oxidative stress, which may alter the effects of insulin and glucose transport (50,51). Moreover, SSAO is functionally identical and coincides with vascular adhesion protein-1 (VAP-1), which is expressed on the luminal surface of endothelial cells and plays a key role in lymphocyte trafficking into the site of inflammation (52). The unique dual action of SSAO/VAP-1 predestinates its potential pathophysiological role in development of type 2 diabetes (53,54). Despite unaltered activity of SSAO under either single intervention in our study the combined challenge associated with its elevation. It remained unclear whether induction of SSAO activity contributed to or resulted from impaired glucose homeostasis. In the same context potential association between SSAO and AOPPs requires further studies.

While AGE-associated fluorescence only tended to increase as a result of combined challenge, levels of non-fluorescent AGE-CML increased significantly. CML is the most abundant AGE in human body, produced particularly via glycoxidation reactions (55). Thus, elevated CML might reflect enhanced oxidative stress imposed by combined dietary challenge. Moreover, CML acts as ligand to RAGE (56) and is elevated in diabetes (53) and implicated in pathogenesis of diabetes and its complications (57). Thus, the role of elevated CML in induction of glucose homeostasis alteration under combined challenge might not be excluded.

The intervention in our study also altered the oxidative status of the offspring. However, these results were not completely consistent, probably because the employed assays estimate different components of antioxidative defense. Moreover, some dietary MRPs possess antioxidant capacity (58). Negative effect of Coca-Cola on TBARS was emphasized by prenatal MRPs load. Lower total antioxidative capacity was visible in both MRP groups, and drinking Coca-Cola even sharpened the differences. On the other hand, Coca-Cola drinking groups had higher FRAP. In previous studies, cola beverages did not affect oxidative or carbonyl stress (47,59). Potential associations between elevated SSAO-induced oxidative stress and observed alterations in oxidative status require further study.

Neither nephrotoxic nor hepatotoxic effects of prenatal and/or postnatal intervention, assessed by urine albumine-to-creatinine ratio and AST activity, were observed.

In conclusion, our results suggested that maternal diet rich in MRPs may adversely affect metabolic status of young-adult female rat offspring and predispose them to higher susceptibility to post-natal Coca-Cola consumption. To verify these findings, there is a need for future studies dealing with the effects in the offspring of both sexes and prolonging the maternal intervention from pregnancy to lactation.
